# Early pandemic impacts on family environments that shape childhood development and health: A Canadian study

**DOI:** 10.1111/cch.13046

**Published:** 2022-09-01

**Authors:** Jessie‐Lee D. McIsaac, De‐Lawrence Lamptey, Jane Harley, Madison MacQuarrie, Randi Cummings, Melissa D. Rossiter, Magdalena Janus, Joan Turner

**Affiliations:** ^1^ Early Childhood Collaborative Research Centre Mount Saint Vincent University Halifax Canada; ^2^ Faculty of Education Mount Saint Vincent University Halifax Canada; ^3^ Department of Child and Youth Study Mount Saint Vincent University Halifax Canada; ^4^ School of Health and Policy Management York University Toronto Canada; ^5^ Applied Human Sciences University of Prince Edward Island Charlottetown Canada; ^6^ Department of Psychiatry and Behavioural Neurosciences McMaster University Hamilton Canada

**Keywords:** child health, COVID‐19, early child development, family, parenting

## Abstract

**Objectives:**

Changes to income and employment are key social determinants of health that have impacted many families during the COVID‐19 pandemic. This research aimed to understand how changes to employment and income influenced family environments that contribute to early childhood development and health.

**Methods:**

A concurrent triangulation mixed method design was used through a cross‐sectional survey on early impacts of the COVID‐19 pandemic involving families with young children in the Canadian Maritime provinces (*n* = 2158). Analyses included multivariate regression models to examine whether changes to employment and income predicted changes to Family access to resources and social support, parenting Abilities and self‐care at home, and home Routines and Environments (FARE Change Scale). Content analysis was used to identify themes from the open‐ended questions.

**Results:**

Changes to employment and income early in the pandemic like no longer working but continued to receive salary, working fewer hours for the same salary earned before the pandemic, no longer working nor receiving salary, working fewer hours resulting in salary reduction, essential worker status and household income were significant predictors of FARE Change Scale when ethnicity/cultural background and province of residence are controlled (*P* < .05). Themes provided a description of family impacts, including shifting employment and income, finding time and capacity, feelings of guilt and the creation of new routines.

**Conclusion:**

Our study provides insight on the implications of public health restrictions, such as the importance of increased time for parents (through reduced work hours) and access to resources and social support to support child development and health.

**Key messages**

- Families experienced abrupt change to employment, income and caregiving responsibilities early in the COVID‐19 pandemic.
- In this study, changes to employment and income were used to explore changes to Family access to resources and social support, parenting Abilities and self‐care at home, and home Routines and Environments (measured through a FARE Change Scale).
- The results provide implications on the impact of public health restrictions that influence child development and health, such as the negative impact of loss of income that could be addressed through government income support programmes.
- Reduced work hours and facilitating access to external resources and social support can also help to strengthen family environments during a time of uncertainty such as a global pandemic.

## 1 INTRODUCTION

The early years of a child's life are a critical period for establishing the conditions for lifelong health. Research consistently demonstrates that early environments profoundly influence physical, emotional, social, cognitive and language development of a child (Black et al., 2017). The COVID‐19 pandemic has altered these early environments for young children through the resulting changes to programmes and services (e.g. healthcare, developmental services, child care, school and recreation), limits to social support and abrupt changes to employment conditions and income for parents (Qian & Fuller, 2020). The Canadian Maritime provinces have been recognized for early implementation of stringent containment measures during the first wave of the pandemic (Rocha et al., 2020), but little is known how the resulting restrictions impacted children and their families. The purpose of this research is to understand how rapid changes to employment and income early in the pandemic have influenced family environments that contribute to early childhood development and health.

### 1.1 Background

**FIGURE 1:**
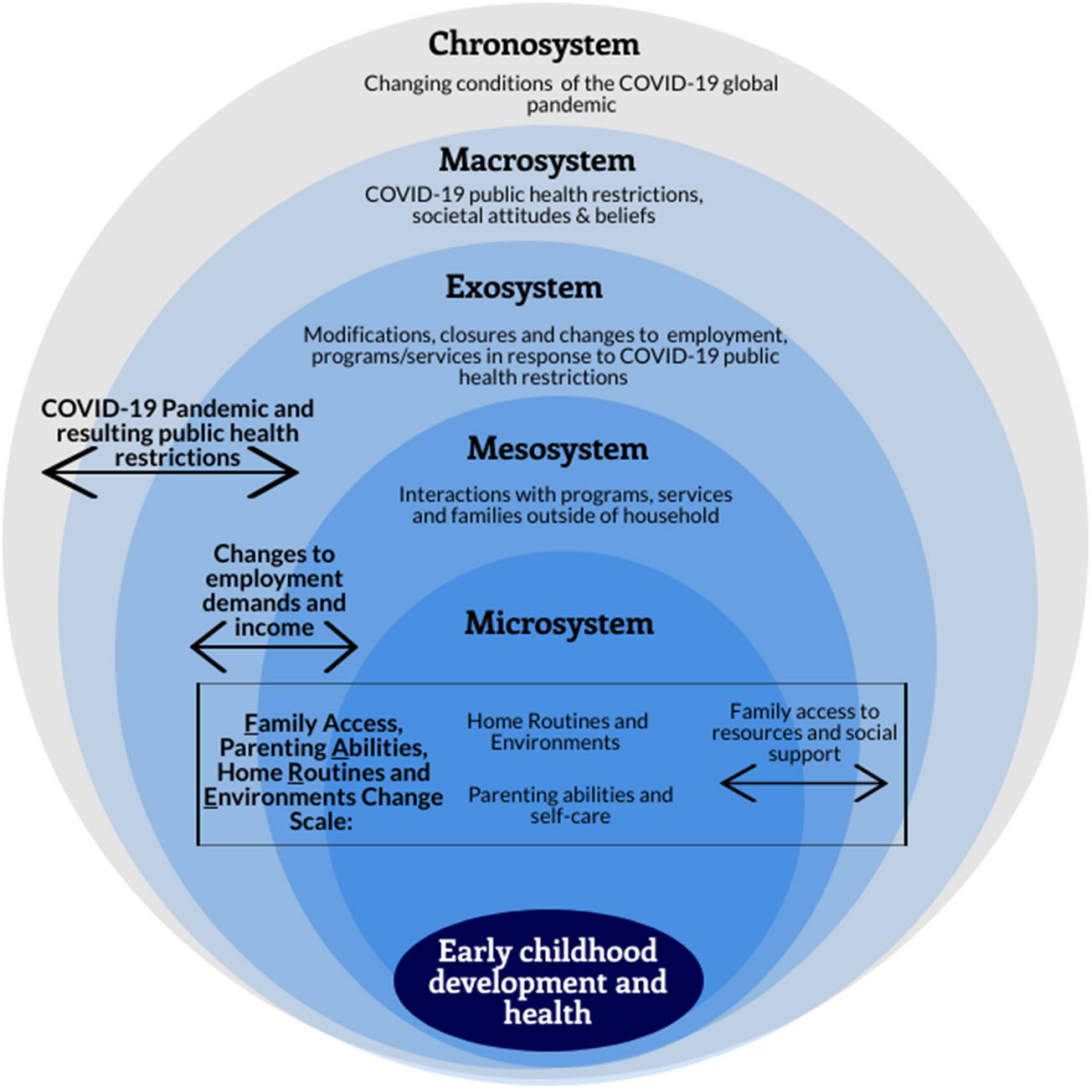
Ecological model with study design concept depicted

Early childhood development is a key social determinant of health (Raphael, 2014). Ensuring access to high‐quality early childhood education, family support and services, and early intervention can improve long‐term health and education outcomes for children and reduce inequities in health, income and education in the population (Black et al., 2017; Marmot et al., 2008). An ecological perspective (Bronfenbrenner, 1977) considers how early childhood development and health is influenced by a system of distal and proximal environments (see Figure 1 for a depiction of the study concept within the ecological model). The chronosystem reflects major societal transitions and events occurring over time, like the COVID‐19 pandemic, that influence attitudes, beliefs and ideals across time and systems. Broader societal and cultural influences at the macrosystem level and specific social structures and policies at the exosystem level related to the pandemic have shaped the environments of families, such as closures of programmes, services and workplaces and reduced access to outdoor public places like parks and playgrounds. Many young children would typically spend a significant amount of time *between* community‐based settings (school, child care, community programmes) and their homes (both microsystem‐level influences). However, initial containment measures during the first wave of COVID‐19 rapidly shifted the focus of early childhood development and health predominantly into the microsystem environment of the family home; as a result, interactions *between* families and community settings were reduced. Therefore, viewing the global pandemic through an ecological systems lens frames family environment at the forefront of public health efforts in the prevention of further spread of COVID‐19 and as potentially the most influential microsystem shaping the development and health of young children (Gadermann et al., 2021).

Canada's unemployment reached record rates during the pandemic as some, and particularly working mothers, experienced abrupt changes to their employment conditions, reduced income and increased caregiving responsibilities with closures to school and child care (Beland et al., 2020; Del Boca et al., 2020; Qian & Fuller, 2020). A focus on family environments emphasizes the importance of family resilience during the pandemic, which may depend on the economic resources of a family as they appraised the situation and rapidly reorganized their environments to make it more manageable (Coleman & Ganong, 2002). Family Resilience Theory turns attention to these adaptation processes of the family that is experiencing significant stress (Patterson, 2002). Understanding the experiences of families early in the pandemic will identify potential supports and stressors during the initial period of adjustment and the social systems, environmental factors and family relational processes that contribute to the family's ability to adapt to adverse events over time (Patterson, 2002).

From an ecological perspective, changes in the macrosystems and exosystems, such as those related to employment conditions and income, are likely to have influenced the microsystem of families' homes and, as a result, their ability to adjust their environments to support children's development and health. For example, *access to resources and social support* was drastically altered among families during the first wave of the COVID‐19 pandemic with initial research suggesting more limited social supports (Roos et al., 2021). Further, healthy, safe and affordable foods as well as family participation in and support for physical activity and play are vital *exosystem* elements that contribute to health‐promoting physical and social environments (Bassett‐Gunter et al., 2017). The pandemic initially led to more time within the family home and were the main environments that shaped a child's nutrition, play and activity. However, the capacity for families to create health‐promoting environments was challenged by stress from job loss and balancing employment demands with fewer resources and social support (Carroll et al., 2020). These challenges may impact *parenting abilities and self‐care*, especially for mothers (Lewis, 2020), who were the parent most likely to balance added responsibilities of child care, supporting play, creating learning opportunities and providing meals at home. High levels of family stress and mental health concerns during COVID‐19 have been reported, which may influence parenting strategies and parent‐to‐child relationships (Prime et al., 2020; Roos et al., 2021). Changes in *home routines* have also occurred as a result of closures of child care and school, which has shaped their *environments* for play, literacy, physical activity, sleep and nutrition. For example, play is essential to enhance children's sense of belonging, confidence and resilience through the mastery of skills, to process stressful events and to share subjective experiences with others (Hertzman, 2010). However, with more isolated time at home, less interaction with peers and fewer community outdoor spaces, children's environments have been impacted by COVID‐19, with early research suggesting lower physical activity levels, less outside time and more sedentary behaviour among Canadian children (Carroll et al., 2020; Moore et al., 2020).

### 1.2 The current study

While studies are emerging to describe the impacts of the pandemic, few have considered the intersection of changes to employment and income across the proximal environments that shape children's development and health. Our study aims to address how the rapid changes to employment and income that occurred early in the pandemic impacted environments that contribute to early childhood development and health in the Maritime provinces. To align with the ecological model (see Figure 1), this research considers the impacts of the COVID‐19 pandemic (chrono–macrosystem level) through investigating how rapid changes to employment and income (exosystem) influenced changes in environments, which was conceptualized through family access to resources and social support (exo–meso–microsystems), parenting abilities and self‐care (microsystem) and home routines and environments (microsystem). The focus on Maritime provinces is novel and important as it is the first study that explored pandemic impacts on the environments that shape Maritime children's development and health. This Maritime study provides insight on the implications of public health restrictions and offers policy recommendations acknowledging the childhood developmental and health needs across systems.

## 2 METHODS

### 2.1 Study design and participants

A concurrent triangulation mixed methods design allowed for a description of family adjustments during the pandemic and the change that families experienced as a result of public health restrictions and protective measures to control the spread. Participants of this study included 2158 families with a child aged 0–8 living in the Canadian Maritime provinces (Nova Scotia, New Brunswick and Prince Edward Island). The survey generated both qualitative and quantitative data to allow for a mixed method design where results were triangulated following independent analysis of each method. The mixed methods approach allowed for an in‐depth exploration of families' environments with the qualitative findings helping to further elucidate the quantitative results. Research ethics approval was obtained from Mount Saint Vincent University.

### 2.2 Data collection

The data for this study were obtained from a cross‐sectional survey study as part of a broader study that was exploring families' adjustments and adaptations throughout COVID‐19. Government and health authority partners contributed to the design of the survey to ensure policy relevance and pilot testing was conducted. The families were recruited through promotion of the survey using social media platforms and by sending recruitment materials through child care and family‐focused organizations. The survey was available online in English early in the first wave of COVID‐19 pandemic when similar lockdown restrictions, including widespread closures to schools and child care, were in place across the Maritime provinces (14 April to 4 May 2020). The survey included both closed and open‐ended questions to explore demographic variables as well as family experiences during the pandemic. In particular, changes to employment and income were assessed with the following variables: changes in hours of employment and remuneration (e.g. work fewer hours resulting in salary reduction and no changes in work demands and salary); doing paid work or school work from home while also balancing parenting responsibilities; employed in a sector considered as an essential service by respective provincial government; and household income. For the purposes of this study, essential services were defined as health; food, agri‐food and fisheries; transportation, including trucking, rail and transit; construction and manufacturing; information technology, telecommunications and critical infrastructure; and public services, such as police, fire and ambulances.

Consistent with our ecological approach, 20 items on the survey focused on family environments that contribute to early childhood development, health and well‐being were combined to form a scale to describe three key areas of change in families: Family access to resources and social support (exo–meso–microsystems), parenting Abilities and self‐care at home (microsystem), and home Routines and Environments (microsystem) (FARE Change Scale; see Figure 1 and Table 1). The scale demonstrated satisfactory Cronbach's alpha level of .76 (*n* = 1769) (Taber, 2018). The scale consisted of 20 items, with each rated on a 5‐point Likert scale: 1 = *much less change*; 2 = *less change*; 3 = *about the same change*; 4 = *more change*; and 5 = *much more change*. The item responses were summed to produce the total scores, which ranged from 1 to 100. A higher score indicated a more favourable rating of family environments in support of early childhood development and health during the pandemic. Open‐ended survey questions were focused on changes and challenges families experienced as a result of closures and physical distancing advice, the impacts families experienced as a result of changes to services and supports, and other services and supports families needed during this time.

**TABLE 1:** The Family access, parenting Abilities, home Routines and Environments Change Scale (FARE Change Scale) generated from the cross‐sectional survey

Items (all rated from 1 to 5)	Valid (%)	M	SE
**Family access to resources and social support**			
My child has time with their friends	1977 (91.61)	1.29	.02
I feel disconnected to my friends/family^a^			
Our family is able to access safe outdoor space	1976 (91.57)	2.86	.02
Our family is experiencing financial difficulties^a^	1976 (91.57)	3.20	.02
Our family is able to access healthy foods	1976 (91.57)	2.98	.01
**Parenting Abilities and self‐care**			
I find it difficult to manage my child's behaviour^a^	1975 (91.52)	3.36	.02
I feel comfortable supporting my child's play	1975 (91.52)	3.09	.02
I feel rested	1975 (91.52)	2.19	.02
I have time to take care of myself	1975 (91.52)	2.10	.03
I have time to prepare healthy meals	1975 (91.52)	3.23	.02
**Home Routines and Environments**			
My child plays independently	1977 (91.61)	3.28	.02
My child plays outside	1977 (91.61)	3.23	.03
My child has easier bedtime and sleep routines	1977 (91.61)	2.67	.02
My child spends time using screens (movies/shows, video games, online resources)^a^	1977 (91.61)	4.16	.02
My child has time with family	1977 (91.61)	3.77	.03
My child has consistent mealtime and snack routines	1977 (91.61)	2.92	.02
Our family plays together	1977 (91.61)	3.85	.02
Our family reads together	1977 (91.61)	3.46	.02
Our family cooks together	1976 (91.57)	3.62	.02
Our family eats together	1976 (91.57)	3.57	.02
**Overall scale** (Cronbach's alpha = .76)	1975 (91.52)	56.97	.18

*Note*: 1 = *much less change*; 2 = *less change*; 3 = *about the same change*; 4 = *more change*; 5 = *much more change*.

^a^ Items are reverse scored.

### 2.3 Data analysis

#### 2.3.1 Quantitative analyses

The quantitative analyses were performed with IBM SPSS Statistics, Version 26.0. Multivariate regression models were used to examine whether changes to employment and income as a result of the pandemic predicted family environments that contribute to childhood development and health. The base model included only variables on changes to employment and income. The full model controlled for ethnicity/cultural background and province. The normal probability plot (P–P) of regression standardized residual included in the analysis showed that the data were normally distributed. The scatterplot also indicated that there were very few outliers that had standardized residuals < −3.3 and >3.3. The Cook distance values were <1 and revealed that there were no influential data points that could potentially lead to a bias model. The correlation among the variables was checked for multicollinearity. The variance inflation factor (VIF) for the variables included in the analysis was less than 10, thus meeting the recommended multicollinearity assumption (Craney & Surles, 2002; Vatcheva et al., 2016). Multiple imputation was used to address data missingness (Madley‐Dowd et al., 2019; van Ginkel et al., 2020).

#### 2.3.2 Qualitative analyses

Qualitative, open‐ended responses from the survey were analysed using a content analysis (Berg & Lune, 2012) approach to openly code and identify emerging themes from participants (1546 participants responded to at least one open‐ended question). Rigour was ensured through two researchers inductively identifying and defining emerging codes with constant comparison to re‐read, re‐code and refine coding as necessary to establish trustworthiness and dependability in the analysis (Berg & Lune, 2012). Themes were established by exploring cross‐case comparisons, identified by patterns and connections both within and across participants.

## 3 RESULTS

### 3.1 Quantitative results

This study included 2158 participants, of which 511 (23.7%) indicated that there were no changes to their employment and income as a result of the pandemic (Table 2). Most participants experienced some form of changes to employment and income during the pandemic had *household income* above $80 000 (*n* = 1012, 60.2%), did *paid work or school work from home while also balancing parenting responsibilities* (*n* = 996, 76.7%) and were employed in a sector considered as an essential service by respective provincial government (*n* = 690, 42.4%). In addition, most participants were mothers (*n* = 1933, 89.6%), between ages 26 and 45 years (*n* = 1766, 92.6%), from European descent (*n* = 1200, 62.9%), had children between the ages of 3 and 5 years (*n* = 1273, 59.0%) and lived in Nova Scotia (*n* = 1419, 65.8%) or New Brunswick (*n* = 552, 25.6%).

**TABLE 2:** Sociodemographic characteristics of participants

Characteristics	Number of cases (*N* = 2158)			
	Valid	Missing	Total	Valid (%)
Changes to employment and income				
Work fewer hours resulting in salary reduction	135	15	150	7.0
Work fewer hours for the same salary earned before the pandemic	302	23	325	15.1
Work more hours resulting in more salary	35	3	38	1.8
Work more hours for the same salary earned before the pandemic	144	5	149	6.9
No longer working but receiving salary	163	10	173	8.0
No longer working nor receiving salary	279	12	291	13.5
No changes in work demands and salary	479	32	511	23.7
Doing paid work or school work from home while also balancing parenting responsibilities	940	56	996	76.7
Employed in a sector considered as an essential service by respective provincial government	643	47	690	42.4
Household income				
Less than $20 000	68	0	68	4.0
$21 000–$40 000	158	0	158	9.4
$41 000–$60 000	215	1	216	12.8
$61 000–$80 000	226	1	227	13.5
$81 000–$100 000	286	0	286	17.0
More than $100 000	726	0	726	43.2
Children's age				
0–12 months	196	22	218	10.1
12–18 months	201	24	225	10.4
19–35 months	517	51	568	26.3
3–5 years	1175	98	1273	59.0
6–8 years	755	66	821	38.0
Relationship with child				
Mother	1784	149	1933	89.6
Father	164	20	184	8.5
Participants' age				
Younger than 19 years	2	0	2	.1
19–25 years	54	0	54	2.8
26–35 years	883	2	885	46.4
36–45 years	881	0	881	46.2
46–55 years	73	0	73	3.8
56 years and above	7	0	7	.4
Parenting arrangement				
Single parenting	154	0	154	8.1
Co‐parenting in same home	1596	2	1598	83.8
Co‐parenting in different homes	108	0	108	5.7
Employment				
Full‐time job	1407	94	1501	69.6
Part‐time job	219	12	231	10.7
Parental leave	155	12	167	7.7
Stay‐at‐home parent/caregiver	162	16	178	8.2
Registered student (full‐time or part‐time)	87	7	94	4.4
Unemployed but looking for work	43	2	45	2.1
Unable to work due to illness or disability	25	0	25	1.2
Ethnicity/cultural background				
Indigenous/Aboriginal	74	0	74	3.9
Acadian	294	0	294	15.4
European	1198	2	1200	62.9
African	70	0	70	3.7
Middle Eastern	35	0	35	1.8
Asian	37	0	37	1.9
Province of residence				
New Brunswick	499	53	552	25.6
Nova Scotia	1307	112	1419	65.8
Prince Edward Island	169	18	187	8.7

The analysis involved five imputations to address data missingness (Graham et al., 2007). The missing values of the total item‐level responses were 5.7%. The regression models were significant (*P* < .001) (Table 3). The models showed that some types of changes to employment and income early in the COVID‐19 pandemic were significant predictors of perceived changes to family environments that support early childhood development and health. The pooled coefficients of the full model (Table 4) demonstrated that scores on the FARE Change Scale significantly increased by 4.01 for participants who were no longer working but still receiving salary; 3.29 for participants who worked fewer hours for the same salary earned before the pandemic; 2.58 for participants who were no longer working nor receiving salary; 2.39 for participants who worked fewer hours resulting in salary reduction; 1.34 for participants whose work demand and salary did not change; and 1.16 for participants whose household income were over 100 000 Canadian dollars. On the other hand, scores significantly decreased by 1.37 for participants who identified as essential workers. On average, the full regression models explained about 6% of the variance observed in scores on the FARE Change Scale (adjusted *R* ^2^ = .06) (Table 3).

**TABLE 3:** Summary of ANOVA results and adjusted *R* ^2^ for the models predicting scores on the FARE Change Scale

Imputation number	Model		Sum of squares	df	Mean square	F	P	Adjusted *R* ^2^
Original data	Base	Regression	3659.50	15	243.97	4.71	<.001	.05
		Residual	54 827.28	1059	51.77			
	Full	Regression	4873.16	23	211.88	4.15	<.001	.06
		Residual	53 613.62	1051	51.01			
1	Base	Regression	7797.459	15	519.83	8.94	<.001	.05
		Residual	120 276.93	2069	58.13			
	Full	Regression	9548.77	23	415.16	7.22	<.001	.06
		Residual	118 525.62	2061	57.51			
2	Base	Regression	6863.23	15	457.55	7.90	<.001	.05
		Residual	119 859.55	2069	57.931			
	Full	Regression	8556.28	23	372.01	6.49	<.001	.06
		Residual	118 166.48	2061	57.35			
3	Base	Regression	8403.08	15	560.21	9.71	<.001	.06
		Residual	119 386.09	2069	57.70			
	Full	Regression	10 128.43	23	440.37	7.71	<.001	.07
		Residual	117 660.74	2061	57.09			
4	Base	Regression	7386.11	15	492.41	8.51	<.001	.05
		Residual	119 719.64	2069	57.86			
	Full	Regression	8869.95	23	385.65	6.72	<.001	.06
		Residual	118 235.80	2061	57.37			
5	Base	Regression	7784.94	15	518.10	9.01	<.001	.05
		Residual	119 229.22	2069	57.63			
	Full	Regression	9582.37	23	416.63	7.31	<.001	.07
		Residual	117 431.78	2061	56.98			

*Note*: The base model included only variables on changes to employment and income. The full model controlled for ethnicity/cultural background and province.

**TABLE 4:** Pooled coefficients (*β*) describing the extent to which changes to employment and income predicted participants' score on the FARE Change Scale

Variables/models	Base model		Full model	
	*β* (SE)	95% CI	*β* (SE)	95% CI
Changes to employment and income (dichotomized for analysis)				
Work fewer hours resulting in salary reduction (yes/no)	2.39 (.73)	.96, 3.82***	2.39 (.73)	.97, 3.82***
Work fewer hours for the same salary earned before the pandemic (yes/no)	3.36 (.59)	2.20, 4.53***	3.29 (.59)	2.12, 4.45***
Work more hours resulting in more salary (yes/no)	−1.20 (1.37)	−3.89, 1.49	−.82 (1.37)	−3.51, 1.89
Work more hours for the same salary earned before the pandemic (yes/no)	−.54 (.78)	−2.06, .99	−.48 (.79)	−2.01, 1.05
No longer working but receiving salary (yes/no)	4.09 (.72)	2.68, 5.51***	4.01 (.73)	2.57, 5.44***
No longer working nor receiving salary (yes/no)	2.68 (.59)	1.53, 3.84***	2.58 (.59)	1.42, 3.74***
No changes in work demands and salary (yes/no)	1.35 (.51)	.35, 2.35**	1.34 (.51)	.33, 2.35**
Doing paid work or school work from home while also balancing parenting responsibilities (yes/no)	.47 (.58)	−.71, 1.65	.528 (.60)	−.72, 1.77
Employed in a sector considered as an essential service by respective provincial government (yes/no)	−1.30 (.45)	−2.19, −.40**	−1.37 (.45)	−2.27, −.47**
Household income				
Less than $20 000 (yes/no)	−1.77 (1.05)	−3.83, .28	−1.74 (1.04)	−3.78, .30
$21 000–$40 000 (yes/no)	.31 (.77)	−1.21, 1.82	.35 (.77)	−1.17, 1.87
$41 000–$60 000 (yes/no)	.48 (.71)	−.91, 1.88	.60 (.72)	−.83, 2.02
$61 000–$80 000 (yes/no)	.25 (.68)	−1.09, 1.59	.46 (.69)	−.91, 1.84
$81 000–$100 000 (yes/no)	−.05 (.64)	−1.32, 1.23	.11 (.66)	−1.21, 1.42
More than $100 000 (yes/no)	.90 (.54)	−.18, 1.97	1.16 (.57)	.03, 2.29*
Ethnicity/cultural background				
Indigenous/Aboriginal descent (yes/no)			−1.40 (.97)	−3.32, .53
Acadian (yes/no)			−1.20 (.48)	−2.14, −.25**
European (yes/no)			−.73 (.43)	−1.60, .14
African (yes/no)			.60 (.96)	−1.29, 2.50
Middle Eastern (yes/no)			−1.61 (1.31)	−4.19, .97
Asian (yes/no)			1.88 (1.27)	−.62, 4.38
Province				
Prince Edward Island (yes/no)			1.08 (.40)	.29, 1.86**
Nova Scotia (yes/no)			1.58 (.62)	.37, 2.78**
Constant	55.12 (.75)	53.63, 56.61***	55.23 (.78)	53.70, 56.76***

*Note*: The base model included only variables on changes to employment and income. The full model controlled for ethnicity/cultural background and province. Beta (*β*) values, standard errors (SE) and 95% confidence intervals (CI) are reported for variables included in each model. No is the reference category for all binary variables.

*** *P* ≤ .001.

** *P* < .01.

* *P* < .05.

### 3.2 Qualitative results

The qualitative findings are presented through four interrelated themes that reflected the impacts of employment and income among Maritime families: shifting employment demands and income loss, difficulty finding time and capacity, feelings of guilt and creating new routines to support a balance in family life.

#### 3.2.1 Shifting employment demands and income loss

Typical routines and family life experienced by Maritime families early in the COVID‐19 pandemic were described through the shifts in employment situations and income loss alongside of the lack of availability of child care and at‐home schooling. Several participants discussed how their employment demands affected the amount of quality time they were able to spend with their children to support their development and health. For participants working from home, the flexibility of the workday, an employer's level of understanding of their child care situation and expectations were sometimes noted: ‘*My work however is not essential and my supervisor and other superiors have been so supportive and accommodating. There is no pressure on me to meet any requirements and to only do what I can do*.’ Other parents indicated less flexibility in their employers expectations: ‘*Our employer keeps telling us to take care of our mental health and our family, but the expectations for our jobs have not lessened*.’ Participants with one or both parents working in an essential service discussed challenges with balancing responsibilities at home. Difficulties finding child care were prominent when both parents were essential workers: ‘*It is incredibly difficult to secure any form of child care when you have a 2‐parent health care worker household. Many see the risk of providing care to our children as too high*.’ Most of the participants who were essential workers had extended family care for their children while they were working.

Some participants described financial hardship as a result of the pandemic, for example: ‘*Both earners in our household lost 100% of their income due to COVID‐19 …. Even with CERB, [Canada Emergency Response Benefit] making our monthly bills is a challenge as we were high income earners and the CERB is only about 25% of our monthly pre‐COVID income*.’ Some families limited their work hours to care for their children but noted the financial limitations associated with this decision, whereas others left their job or took unpaid leave to care for their children. In some families, the pandemic caused participants to lose their jobs or experience decreased hours, resulting in financial hardship. Others reported limited or no financial difficulties, with some indicating they experienced lessened financial stress without the added costs of child care during closures.

#### 3.2.2 Difficulty finding time and capacity

Participants indicated that balancing employment, parenting and other household duties created long days for parents and resulted in limited time for self‐care. Experiences working at home were intertwined with the reality of little to no separation or schedule for work and parenting responsibilities. Participants described employment priorities as competing with increased parenting and household responsibilities with some expressing that it was unsustainable, for example: ‘*Prior to COVID, I worked an average of 40+ hours per week, with a good workload. Now I'm trying to complete the same quality of work with twice the distractions and added school work to keep on top of everything*.’ Many participants talked about the challenges of being responsive to their child's needs throughout the day and referred to this as ‘*entertaining*’ their children or keeping them ‘*busy*’. The loss of routine was a challenge for many children, as described by one participant: ‘*My children respond well to routine and the structure school provides. The lack of routine due to conflicting parent work schedules is reflected negatively in their behaviour*.’ Another participant explained their struggles to provide their child with enough stimulation to support their child's development: ‘*The pressures my wife and I feel to juggle our work with family responsibilities makes it difficult to have adequate energy and time to be providing them ideal stimulation*.’ Further, new demands related to at‐home learning for school‐aged children required parents to navigate online supports, services and activities. Participants discussed their concern for their child's development and progress in school as many noted that they are not trained as teachers, for example: ‘*Trying to keep up with my six‐year old's French homework is also challenging. We're not a French household and I'm trying to teach him French and keep him from regressing too much. It's hard*.’

#### 3.2.3 Feelings of guilt

Participants discussed the uncertainty of how the pandemic would influence their family over a longer term and the choices they made to balance their employment and parenting responsibilities: ‘*But if you spend lots of extra parenting time then you feel the guilt later on for not being as productive at work so I try to compensate with extra hours. So, in the end I'm working longer hours than normal and just really tired at the end of the day*.’ Some families also reported less access to outdoor spaces due to the pandemic closures and that their work schedules did not leave much time for supervising children outside. Parents expressed their guilt about the increased screen time for children, especially when they compared it with their typical physical activity and outdoor time at child care and/or school, for example: ‘*I worry so much about my daughter having to spend so much time alone and so little time being active outside as all we can do is walk around the block when I'm able to take her as she cannot go for a walk unsupervised*.’ Finally, when child care was accessed outside of the home, it was most frequently provided by grandparents. Parents reported that this allowed them to work uninterrupted but also led to concerns about the spread of the virus to both their family members and their children. Some essential workers who involved extended family in caregiving expressed worry but felt that this was their only option: ‘*My husband is a [health care worker] and we have a newborn. We were worried about him making the newborn sick so he moved out. For the last 7 weeks I have essentially been a single mother to a newborn and a toddler while still trying to maintain a few [work] files. It has been extremely challenging*.’

#### 3.2.4 Creating new routines to support a balance in family life

With the loss of external supports, such as child care and school, participants reported how they adjusted to family routines to balance employment, parenting and household responsibilities. Some participants indicated that their changing employment demands allowed them flexibility to work at different times to balance other responsibilities. For two‐parent households, many discussed strategies where spouses coordinated their time based on job demands to take turns with parenting and other household tasks: ‘*My wife and I have condensed our work days to split child care so our boys have one parent with them all the time throughout the week*.’ Further, some participants expressed that losing employment allowed them greater time with their children during the initial closure to allow for broader learning opportunities such as through crafts, baking and gardening. Families also adjusted to new routines with some participants indicating their enjoyment with new roles: ‘*I love having my son home with me. I love being his teacher and him having one‐to‐one support in his learning*.’ The adoption of new routines allowed families to experience a similar or new sense of normalcy amidst the many changes.

## 4 DISCUSSION

Families in this study described the perceived impact on child development and health as a result of changes to employment demands and income that shaped different home routines and environments. Our study demonstrated that families of children under 9 years in the Maritime provinces of Canada across employment situations expressed feelings of guilt and uncertainty as they navigated their new realities and responded in the best way that they could with the available resources in their homes, neighbourhood and broader community. It is important to note that the stressors and demands were not experienced similarly by all families during the COVID‐19 pandemic. For example, our regression results suggest that having more time available during the pandemic (through reduced working hours) was a positive change to the family environment, even if there was less overall income through a reduction to salary. Our qualitative results revealed that some families preferred to limit their work hours, leave their job or take unpaid leave to care for their children despite reduction to salary. For these families, the uncertainty during the first wave of the pandemic was concerning and disruptive; having additional time to care for their children and themselves seemed to be a positive change early in the pandemic. However, we did observe that other families affected by income loss or reduction, and essential workers experienced changes in routines that tended not to be easy to address through reorganizing routines within the home when access to resources and social support was unavailable. Further, while families reported it was difficult to manage multiple demands, the tendency was towards positive appraisal of the situation and creation of favourable home environments and routines for child development and health.

From the perspective of family resilience (Patterson, 2002), the experience of families with young children during the pandemic can be described through the appraisal and reorganization process that has been required due to the restructuring of external resources and social supports. Families in this study specifically spoke about the difficulty to find time and capacity to prioritize spending time supporting their child's physical and social emotional health and development while they balanced competing responsibilities, which resulted in feelings of guilt. Recent research has discussed the unprecedented demands of parenthood with increased household and family responsibilities and, at the same time, less access to supports (Evans et al., 2020). A gender imbalance suggesting a greater privileging of men's work has also been reported as families have coordinated employment and household tasks (Manzo & Minello, 2020). In Canada, labour force data have suggested a gender employment gap among parents with young children (Qian & Fuller, 2020), while a study has reported increased participation in housework and child care by fathers during the pandemic (Shafer et al., 2020). Maritime families in this study were primarily represented by mothers; many families were also employed full‐time, which provided context to the types of experiences that were shared, which is important considering the recognition of gender as a social determinant of health. However, future research is needed to further elucidate differential impacts of COVID‐19 through a more economically diverse and gendered balanced group of respondents.

The impact of the changes to parenting and children's environments on their development and health is not yet well understood. Many parents in this study indicated that working from home indicated that it was difficult to balance employment demands and facilitate health‐promoting activities such as outdoor play and physical activity. As a result, parents were concerned about the increased screen time and sedentary behaviours among their children, which aligns with the results of previous studies (Carroll et al., 2020; Moore et al., 2020). Another study described broader impacts of school shutdown on children's health, through food behaviours and sleep (Mantovani et al., 2021). Our study further extends existing research by considering access to resources and social support and parenting abilities. Past studies of global environmental events, such as floods and hurricanes, have discussed the importance of social support and cohesion as a protective factor in families' adaptation to their new realities (Greene et al., 2015; Reid & Reczek, 2011; Sutherland & Glendinning, 2008). In contrast, the initial containment measures of COVID‐19 led to greater isolation of families with a loss of typical social support structures as a result of closures to programmes and services alongside of restrictions to gathering limits and social circles. As the COVID‐19 experience of families is indeed unique and is continuing to have ongoing impacts, continuing to explore family resilience over time will be important to identify the external supports that are required to support positive adaptations.

An ecological system perspective considers impacts arising from developments across the chronosystem, such as those that relate specifically to the COVID‐19 pandemic. Ultimately, the impacts within and across system levels play a profound role in shaping a child's environment and influencing their overall development and health (Hertzman, 2010; Raphael, 2014). The impacts of changes to income and employment, at a more proximal environment, are considered for their impact on childhood development and health in this study. The initial containment measures led to families spending more time within the home environment (microsystem) without access to child care and school and, as a result, fewer interactions (mesosystem) with programmes and services that are typically available that could support a child's development and health. While research is beginning to describe the reported high levels of family stress and mental health concerns during COVID‐19 (Prime et al., 2020; Roos et al., 2021) and impacts on health behaviours (Carroll et al., 2020; Moore et al., 2020), this study provided a unique contribution through its consideration of the intersection between changes to employment and income across the proximal environments that shape children's development and health.

### 4.1 Strengths and limitations

This mixed methods research allowed for triangulation of quantitative and qualitative data from an online survey to describe how employment and income influenced the environments that shape early childhood development and health early in the COVID‐19 pandemic. We focused on family employment situations considering the significance during COVID‐19, such as the implications to families that were balancing working from home (paid work and school work) and those who were considered to be an essential worker. More studies are needed to provide a deeper understanding of other important factors that may contribute to changes in family life as a result of COVID‐19. Despite our concerted efforts to make the survey available to all Canadian Maritime families with children aged 0–8 years, most of the participants were mothers, co‐parenting with their spouses or partners in the same home with household income above $80 000, had full‐time jobs, were from European descent and lived in Nova Scotia or New Brunswick. More research is needed to better understand the perspectives of fathers and the experiences of families from more diverse backgrounds (e.g. single parents, low‐income households and ethnic/racial minorities), which can provide policymakers and administrators with critical directions for addressing the unique needs of all families. Further, more research is needed to better understand the experiences of families living in other provinces in Canada. Although the FARE Change Scale we generated from the survey obtained satisfactory reliability (Cronbach's alpha = .76), the scale lacked criterion validity. More research is needed to further validate this scale and build on this preliminary understanding of the impact of changes to employment and income on family environments to support their children's development and health early in the COVID‐19 pandemic. The full regression models generally explained a small amount of the variance (adjusted *R* ^2^ = .06) based on Ferguson's (2009) criteria of .04 = recommended minimum effect representing a ‘practically’ significant effect for social science data, .25 = moderate effect and .64 = strong effect (see Table 3). This study provides only a preliminary understanding of the intersection of changes to employment and income across proximal environments that shape children's development and health in the context of the early stages of the novel COVID‐19 pandemic when knowledge about the behaviour of SARS‐CoV‐2 was very limited and families had to adjust to sudden changes to their way of life as a result of strict public health measures in the region that drastically modified their environments.

## 5 CONCLUSION

This study describes how changes to employment and income influenced family environments that contribute to early childhood development and health among families in the Maritime provinces in Canada. Families found it difficult to manage the abrupt changes to work demands, income and reduced access to external supports during the first wave of COVID‐19, yet many were able to respond through the creation of new routines to meet the needs of their family. The results inform the development of potential supports in the mitigation of similar stressors into the future. As well as directing support to families who have lost income during the pandemic (i.e. through government income support programmes), policymakers should consider the potential support that is offered through increased time (through reduced work hours) and access to external resources and social support that can strengthen family environments during a time of upheaval and uncertainty. Our study has policy significance for public health efforts to alleviate the impacts of future sudden events that rapidly change children's immediate environments. To further understand how environments during the pandemic have shaped children's health, research should explore families' resilience throughout the pandemic. As governments and public health continue to respond to the evolving situation caused by this pandemic, implications of public health restrictions on families with young children demonstrated by this study can inform ongoing decision making related to COVID‐19 and other disruptive events.

## CONFLICTS OF INTEREST

The authors have no conflicts of interest to declare that are relevant to the content of this article.

## ETHICS APPROVAL

All procedures performed in studies involving human participants were in accordance with the ethical standards of the institutional and/or national research committee (Mount Saint Vincent University Research Ethics Board, 2019‐183) and with the 1964 Helsinki declaration and its later amendments or comparable ethical standards.

Informed consent was obtained from all individual participants included in the study.

## AUTHOR CONTRIBUTIONS

Research funding was obtained by JDM and JT for the broader research project. All authors contributed to the study conception and design. The manuscript was drafted by JDM, DL and MM with contributions from JH, RC, JT, MJ and MDR. All authors critically revised the manuscript, and all read and approved the final manuscript.

## Data Availability

The data that support the findings of this study are available on request from the corresponding author. The data are not publicly available due to privacy or ethical restrictions.
